# Progress with COVID-19 vaccination in the WHO African Region in 2021

**DOI:** 10.11604/pamj.supp.2022.41.2.34102

**Published:** 2022-05-02

**Authors:** Balcha Masresha, Alain Poy, Goitom Weldegebriel, Selemani Mbuyita, Daniel Fussum, Ado Bwaka, Gilson Paluku, Phionah Atuhebwe, Richard Mihigo, Benido Impouma

**Affiliations:** 1 WHO Regional Office for Africa, Brazzaville, Congo,; 2 WHO Inter-country Support Team for Eastern and Southern Africa, Harare, Zimbabwe,; 3 WHO Inter-country Support Team for Western Africa, Ouagadougou, Burkina Faso,; 4 WHO Inter-country Support Team for Central Africa, Libreville, Gabon

**Keywords:** COVID-19 pandemic, vaccines, vaccination coverage, Africa

## Abstract

**Introduction:**

as of end 2021, ten different vaccines have received Emergency use listing by the World Health Organisation. The vaccination response to the COVID pandemic started in February 2021 in the WHO African Region. WHO proposed a national coverage target of fully vaccinated population of 40% by the end of December 2021. This manuscript attempts to review the progress in the roll-out of COVID-19 vaccination in the African Region.

**Methods:**

we analysed the aggregate COVID-19 vaccine uptake and utilization data from the immunisation monitoring databases set up by countries and shared with the WHO Regional Office for Africa.

**Results:**

as of 31 December 2021, a total of 340,663,156 doses of COVID-19 vaccine were received in 46 countries in the African Region. The weekly average doses administered was 4,069,934 throughout the year. In the same period, a total of 114,498,980 persons received at least one dose, and 71,862,108 people were fully vaccinated, amounting to 6.6% of the total population in the Region. Only 5 countries attained the target of 40% full vaccination coverage. Disaggregated information was not available from all countries on the number of persons vaccinated by gender, and according to the priority population groupings. A total of 102,046 cases of adverse events following immunisation (AEFIs) were reported among which 6,260 (6.1%) were labelled as severe AEFIs.

**Conclusion:**

COVID-19 vaccination coverage remains very low in the African Region, with all but 5 countries missing the 40% coverage target as of December 2021. Countries, donors and partners should mobilise political will and resources towards the attainment of the coverage targets. Countries will need to implement vaccination efforts using tailored approaches to reach unreached populations. The reporting gaps indicate the need to invest on efforts to improve the capture, analysis and use of more granular program data.

## Introduction

COVID-19, is a respiratory infection that was first discovered on December 2019, in Wuhan city, Hubei Province, China. It is caused by a novel coronavirus called Severe Acute Respiratory Syndrome Coronavirus 2 (SARS-CoV-2). On 30^th^ January 2020, the World Health Organization (WHO) declared the outbreak a Public Health Emergency of International Concern. By 11^th^ March 2020, COVID-19 was declared a pandemic disease [1]. By the end of 2021, a total of 7,262,708 confirmed COVID cases and 156,290 deaths have been reported in the countries in the WHO African Region [2]. Since the declaration of the pandemic, global efforts were directed in four main areas: mobilizing resources and strengthen health system capacities for preparedness and readiness, putting efforts in detection, protection and treatment, reduce transmission and investing in innovation while learning [3]. Technological investments, health system capacity strengthening and socio-economic and social-cultural measures were all taken simultaneously [4].

In the acute phase of the pandemic, WHO provided significant leadership to guide the world towards adaptation and implementation of public health and social measures. These measures played a critical role to suppress SARS-CoV-2 transmission and reduce mortality and morbidity from COVID-19 in many places around the world [5]. Such measures included personal protective measures (e.g. physical distancing, avoiding crowded settings, hand hygiene, respiratory etiquette, mask-wearing); environmental measures (e.g. cleaning, disinfection, ventilation); surveillance and response measures; physical distancing measures; and international travel-related measures [6]. The development of COVID-19 vaccines has been expedited and currently, there are 10 vaccines that have received WHO Emergency Use Listing [7]. As of 31 December 2021, there are 137 types of vaccines in clinical development and 194 more in pre-clinical development stages [8]. On 31^st^ December 2020, the WHO listed the first COVID-19 Vaccine under the Emergency Use Authorization since the outbreak began - Comirnaty COVID-19 mRNA vaccine from Pfizer/BioNTech [9].

Forty-six of the 47 countries in the African Region of the WHO developed their National Vaccine Deployment Plans (NDVPs) for COVID-19 vaccination. The NDVPs outlined the target populations and resources required for phased vaccination. The phasing approach was proposed by the WHO Strategic Advisory Group of Experts (SAGE) to protect the most vulnerable as a matter of priority [10]. In developing the NDVPs, countries adopted their own prioritization based on their contexts, and selected the operational approaches for service delivery. The COVAX facility was established to support eligible countries to gain access to COVID-19 vaccines by mobilizing resources and organizing advanced market commitment arrangements with pharmaceutical companies, among its other mandates and functions. On the other hand, the African Union and Africa CDC set up a purchase facility called African Vaccine Acquisition Trust (AVAT), to enable African countries to access vaccines from the international market [11,12]. At the same time, countries have organized their own direct purchase or received vaccines from other countries through bilateral negotiations and donations. The first shipment of COVID-19 vaccines arrived in the African Region in February 2021.

WHO proposed a national coverage target of fully vaccinated population of 10% by the end of September 2021, 40% by the end of December 2021 and 70% by June 2022 for all countries [13]. As of 31 December 2021, a total of 3,027,874,969 persons have received at least one dose worldwide, and 1,840,432,282 persons have been fully vaccinated, amounting to 23% of the global population [5]. About one year since the first COVID-19 vaccine was listed for emergency use, countries have acquired significant experience in utilizing COVID-19 vaccines with various degrees of coverage. With the geographical and contextual differences, such experiences tend to differ from one country to another. While many studies have analyzed and documented vaccine use and public perceptions, such documentations and analyzes remain limited to smaller contexts. This manuscript attempts to review the progress in the roll-out of COVID-19 vaccination in the African Region.

## Methods

**Study design:** we conducted a descriptive study on the progress of the introduction of COVID-19 vaccines in the countries of the African Region. The WHO African Region consists of 47 countries. The study included countries in the WHO African Region which have received any number of doses of COVID-19 vaccines and have started implementing a COVID vaccination program as of the end of December 2021. Thus, 46 out of the 47 countries of the African Region were included for the analysis.

**Data sources and variables:** all data used to conduct the analysis, interpretation and draw learning are longitudinal secondary data originating from the member states of the WHO African Region. With the launch of COVID-19 vaccination, countries set up monitoring systems to track the delivery of services, building on and integrated in existing immunisation service monitoring systems. In some countries, the information systems were linked with the District Health Information Systems (DHIS-2) platform. Countries regularly updated these datasets and shared with the WHO Regional Office for Africa, which aggregated the data into an online dashboard. We also reviewed a detailed case-based reporting database for Adverse Events Following Immunisation (AEFI), which captures the clinical picture of AEFI cases, including demographic characteristics, dates of onset, date of investigation, symptoms, type of vaccine and outcome.

**Variables and variable definitions:** an explicit selection of variables of priority was made. Primary variables were those being used by WHO to provide the status of global indicators for COVID-19 vaccination. These included the number and type of doses of COVID-19 vaccine received by countries, the number of doses of vaccine administered, the number of persons who received at least one (first) dose vaccine, the number of those who are fully vaccinated, and the number of reported Adverse Events Following Immunization (AEFI), among others. Other indicators include the disaggregated number of persons among various groups who were vaccinated, including breakdown by gender, health workers, persons with comorbidities, displaced populations.

In the COVID-19 vaccination program and in the context of this paper, we defined the above variables as follows: number of vaccines received by a country was defined as a total amount of COVID-19 vaccine doses of any type that a country has received from any source. Number of doses of vaccines administered was defined as a total of COVID-19 vaccines by type that have been inoculated in eligible persons (based on country lower age limit). Coverage with at least one dose of COVID-19 vaccine is defined as the proportion of persons who received a first dose of any COVID-19 vaccine out of the total population. Full vaccination was defined as receipt of 2 doses of AstraZeneca, Gamaleya, Sinopharm, SinoVac, Pfizer-BioNTech, Moderna vaccine or 1 dose of Janssen COVID-19 vaccine. For purposes of calculating coverage, we used the total population estimates based on the UN Population Division (UNPD) projections for 2021 [14].

**Data analysis and interpretation:** we analysed the aggregate vaccine uptake and utilization data submitted by countries to the WHO Regional office for Africa using an online data entry platform [15]. Countries started reporting data to the WHO Regional office for Africa (WHO AFRO) on 17 March 2021. Our analysis covered the period up to 31 December 2021. The analysis was preceded by development of an analytical plan which took into account the main question to be answered from the data and the rationale for conducting the analysis. It also included listing all databases that were to be used as data sources, creating a working copy of the dataset, compare existence of required variables from all sources across the countries.

The trend analysis of the main variables constituted the main dimension of data analysis. Other dimensions of the analysis included comparisons by country, time periods of implementing COVID-19 vaccination, disaggregation by age, gender and subpopulations constituting vaccine recipients. Data analysis was mainly based on the generation of tables, charts and graphs from the databases. We analyzed the frequency statistics using MS Excel. Data interpretation was conducted in a series of virtual discussion sessions among the authors.

## Results

### COVID-19 Vaccine supply in the African Region

As of 31 December 2021, a total of 340,663,156 doses of COVID-19 vaccine were received in 46 countries in the African Region. In the month of December alone, a total of 91,952,058 doses were received, amounting to 27% of all vaccine doses received over the course of the year. Our analysis of sources of the vaccines showed that, more than a half of the vaccine doses (199,952,377 doses) were procured through the COVAX facility (59%). Donations and purchases through bilateral agreements accounted for 96,465,109 doses (28%) while AVAT provided 22,465,790 doses (7%). Six percent of doses were not associated with any specific sources.

Analysis by type of vaccines showed that AstraZeneca constituted the highest proportion (23%) of the vaccine doses received in the Region. Other types of vaccines included Janssen (19.3%), Sinopharm (18.7%) and Pfizer (17.6%) (Table 1). On average, each of the 46 countries received 4 types of vaccines. The minimum number of types of vaccines in a country was 2 (in Zimbabwe) and the maximum was 7 (in Guinea) followed closely by Uganda and Ghana which received 6 types of vaccines each. More than a half of the countries (twenty-seven countries) received more than 3 types of COVID-19 vaccines since the beginning of the vaccine response to the pandemic. The average weekly receipt of COVID-19 vaccines has seen a sharp spike from around 2 million doses per week in June and July 2021 to 7.7 million in August 2021, and steadily increasing to an average of 23 million per week by December 2021. The weekly average doses administered was 4,069,934 throughout the year, but this average increased from 1.4 million per week from March - July to 9 million per week in December (Table 2).

**Table 1:** volumes and types of vaccines received in the WHO African region, 2021

Type of vaccine	Number received	Percentage
AstraZeneca	78,212,520	23.0%
Janssen	65,688,507	19.3%
SinoPharm	63,711,370	18.7%
Pfizer	59,876,630	17.6%
SinoVac	36,659,114	10.8%
Moderna	19,307,500	5.7%
Gamaleya	2,379,440	0.7%
Covaxin	235,000	0.1%
Unknown	14,593,075	4.3%
**Total**	**340,663,156**	**100%**

**Table 2:** weekly average doses of vaccine received and administered by month. WHO African Region, 2021

Month	Weekly average vaccine doses received in countries	Weekly average vaccine doses administered
March	6,768,677	923,538
April	767,670	1,185,993
May	1,114,190	1,047,968
June	1,994,006	1,910,736
July	2,063,605	2,015,962
August	7,699,538	3,870,348
September	9,389,678	6,959,801
October	12,536,324	5,794,758
November	16,383,175	7,313,983
December	22,988,015	9,048,154

### The trend of COVID-19 vaccination in the African Region

**Figure 1:**
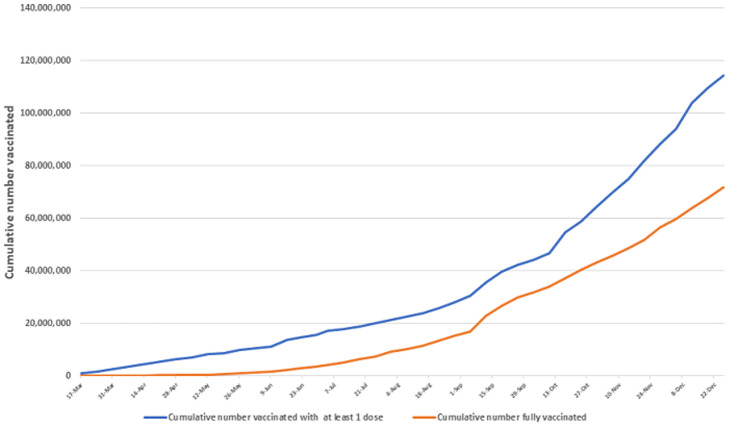
cumulative number of persons vaccinated in the WHO African Region Data as of 31 December 2021

As of the end of 2021, a total of 179,289,703 doses of COVID vaccine were administered across the Region, constituting 53% of the doses received. In the same period, a total of 114,498,980 persons received at least one dose, and 71,862,108 people were fully vaccinated. The total number of people fully vaccinated corresponds to 6.6% of the total population in the Region (Figure 1). Countries have been expanding the target groups for COVID-19 vaccination. By December 2021, twenty-eight countries were providing COVID-19 vaccination to all persons aged 18 years and above, while 15 countries widened their vaccination target to all persons aged 12 years and above. By the end of year 2021, five countries (Seychelles, Rwanda, Mauritius, Cape Verde and Botswana) attained 40% full vaccination coverage rates, while 21 countries have surpassed 10% full vaccination coverage (Table 3).

**Table 3:** COVID-19 vaccination and coverage levels by country, December 2021

Country	Total population	Total number of COVID vaccine doses administered	Number of persons fully vaccinated	% population fully vaccinated
Angola	33,933,610	11,039,082	3,836,693	11.3%
Benin	12,451,040	1,773,592	1,319,264	10.6%
Botswana	2,397,241	1,169,197	1,033,971	43.1%
Burkina Faso	21,497,096	1,053,330	649,320	3.0%
Burundi	12,255,433	6,421	4,453	0.04%
Cabo Verde	561,898	571,130	255,954	45.6%
Cameroon	27,224,265	1,020,007	659,641	2.4%
Central African Republic	4,919,981	518,577	426,546	8.7%
Chad	17,305,108	330,522	93,563	0.5%
Comoros	888,451	581,547	246,139	27.7%
Congo	5,657,013	758,891	583,609	10.3%
Côte d'Ivoire	27,053,629	7,113,233	2,162,458	8.0%
Democratic republic of Congo	92,377,993	333,589	117,844	0.1%
Equatorial Guinea	1,449,896	452,572	203,357	14.0%
Eritrea	3,601,467	0	0	0.0%
Eswatini	1,172,362	401,306	302,304	25.8%
Ethiopia	117,876,227	10,916,152	3,988,780	3.4%
Gabon	2,278,825	438,557	191,549	8.4%
Gambia	2,486,945	280,797	235,633	9.5%
Ghana	31,732,129	7,755,231	2,334,010	7.4%
Guinea	13,497,244	2,823,085	1,018,948	7.5%
Guinea-Bissau	2,015,494	413,938	23,144	1.1%
Kenya	54,985,698	9,723,787	4,018,791	7.3%
Lesotho	2,159,079	698,383	735,610	34.1%
Liberia	5,180,203	880,313	755,263	14.6%
Madagascar	28,427,328	742,069	541,160	1.9%
Malawi	19,647,684	1,637,998	654,927	3.3%
Mali	20,855,735	1,071,777	400,589	1.9%
Mauritania	4,775,119	1,863,548	734,881	15.4%
Mauritius	1,273,433	2,036,896	909,900	71.5%
Mozambique	30,066,648	14,408,516	5,924,305	19.7%
Namibia	2,587,344	632,244	334,702	12.9%
Niger	23,591,981	1,716,942	963,375	4.1%
Nigeria	211,400,708	14,838,199	4,482,899	2.1%
Rwanda	13,276,513	12,588,963	5,435,180	40.9%
Sao Tome and Principe	223,368	137,612	51,216	22.9%
Senegal	17,196,301	1,939,324	944,643	5.5%
Seychelles	98,908	183,472	78,263	79.1%
Sierra Leone	8,141,343	923,880	385,232	4.7%
South Africa	60,041,994	27,802,711	15,763,964	26.3%
South Sudan	13,381,378	268,640	181,577	1.4%
Togo	8,478,250	2,413,619	1,020,872	12.0%
Uganda	47,123,531	9,763,030	1,408,222	3.0%
Tanzania	61,498,437	2,431,769	1,359,225	2.2%
Zambia	18,920,651	1,041,441	651,965	3.4%
Zimbabwe	15,092,171	7,248,458	3,129,823	20.7%

The gender breakdown of fully vaccinated persons is available only from 24 countries, and it shows that women make up 49% of the total. However, country by country disaggregated analysis shows that the proportion of women among the vaccinated ranges from 28% in Gabon to 59% in Lesotho, with 16 countries reporting less than 50% women out of all fully vaccinated. Three countries (Cameroon, Chad and Gabon) reported less than 40% women out of the total fully vaccinated persons. Information on the vaccination of health workers was available from only 31 countries where 2,672,932 health workers received at least one dose vaccine, and 1,882,286 were fully vaccinated in 28 countries. Twenty-two countries have reported a total of 7,239,379 persons with co-morbidities who have been vaccinated with at least one dose COVID-19 vaccine, and 20 countries reported 4,517,705 fully vaccinated persons in this group. The number of fully vaccinated older adults is 6,051,595 as reported from 25 countries. Only 5 countries reported the number of refugees and displaced persons vaccinated. Countries known to host a large number of refugees and displaced persons like Ethiopia, South Sudan, Uganda did not report on this parameter.

### Monitoring of adverse events following immunization

A total of 102,046 cases of adverse events following immunisation (AEFIs) were reported by 31^st^ December 2021 as part of the aggregate reports to WHO AFRO. Out of these 6,260 (6.1%) were labelled as severe AEFIs. The proportion of severe AEFIs range from <5% in 31 counties, to more than 15% in the Gambia, Senegal and South Africa. The rate of reporting of AEFI cases per 100,000 doses provided is highest in Burkina Faso (447 per 100,000) and lowest in Mauritania (at 1.2 per 100,000 doses). The median rate is 45 per 100,000. Thirteen countries have rates in excess of 100 AEFIs per 100,000 doses. The rates of serious AEFIs reported in the aggregate reporting system range from 0 in Kenya to 316 per million doses administered in the Central African Republic. The median rate is 14 per million doses, and the average is 35 cases of serious AEFI per million doses provided.

The detailed information of 34,034 cases of AEFI were reported from across the Region through the Regional COVID-19 vaccination AEFI database. The most common symptoms reported are headache (17%), fever (13%) and muscle or joint aches (10%). Local reactions (soreness, swelling, pain or itching at site of injection) were noted in 7% of cases. There were 178 cases of anaphylaxis reported, of which 52 (29%) were from South Africa, 43 (24%) from Nigeria and 41 (23%) from Togo. Ninety-eight (57%) of these cases were reported following the administration of AstraZeneca vaccine, while 37 (22%) followed the administration of Pfizer vaccine. The database included 33 cases of thrombotic events. Only three deaths were reported in the database among the AEFI cases.

## Discussion

COVID-19 vaccination was initially introduced in most countries in the African Region using AstraZeneca vaccine. The introduction process in many countries was rushed, since countries were dealing with new vaccines that have a very brief shelf-life at the time of receipt. Moreover, the shipment of AstraZeneca vaccine from the Serum Institute of India was suspended when India started experiencing a huge wave of COVID-19 transmission [16]. This directly affected the provision of second dose vaccines in many countries. The overall availability of vaccines improved with multiple countries committing donations starting in mid-August 2021. The sense of urgency continued with supplies of the Pfizer and Moderna vaccines (mRNA vaccines), which have even more stringent cold chain requirements.

The proportion of doses administered in the Region by the end of the 2021 was only 53%, reflecting the increasing volumes of doses that were received in the last weeks of the year, and the relatively slow rate of implementation in many countries. We also note the multiple types of COVID-19 vaccines in countries, with some countries receiving 6 or more types of COVID-19 vaccine products. This has been linked to challenges with logistics, training, communication, service delivery and monitoring [17]. The initial plan and approach in many countries were to target priority populations in a phased manner. However, within a few months, national COVID-19 vaccination coordinating taskforces made decisions to provide the vaccines to wider population groups than stipulated in the national plans.

COVID-19 vaccination coverage across the African Region shows that the majority of countries are not yet at 5% full vaccination coverage, while only 5 countries have met the proposed 40% target for December 2021. We note that, as of end of 2021, a total of 114,498,980 persons received at least one dose, while nearly 72 million people were fully vaccinated in the African Region. To attain the proposed 70% coverage target for June 2022, the countries in the Region will need to fully vaccinate an additional 689 million persons, which means that the average weekly rate of vaccination has to scale up more than six-fold from the current 4 million to 26 million per week throughout the first half of 2022. Even a more realistic target of 50% will require that countries reach 18 million persons per week in the next 6 months to reach an additional 459 million people.

After the first few months of accelerated vaccine deliveries to African countries, COVAX and AVAT issued statements to donor nations at the end of November 2021, requesting them to release donated doses in large volumes and in a predictable manner; that donated doses should have a minimum of 10 weeks shelf life when they arrive in-country, and that recipient countries should be given early notice before the planned arrival of vaccines in-country. There have also been calls for ensuring that syringes and diluents accompany donations [18]. This latter point is the principle of bundling which is widely used in organizing mass vaccination campaigns [19]. Developed countries have already made significant progress with delivering full vaccination to their populations and are even rolling out booster doses [5-7]. With the increasingly wide discrepancies in vaccine coverage rates, the issue of vaccine equity is taking center stage [20-22]. Unfortunately, a slow and delayed vaccination rollout in the developing world will pose a risk for the emergence of COVID-19 variants and recurrent waves of intense transmission with subsequent loss of lives and livelihoods.

Israel and Bhutan attained high vaccination coverage within a short period of time after securing large number of doses within a short period of time. Bhutan received two consignments of vaccine doses in March 2021, adequate to vaccinate all eligible adults in the country [23]. Rosen *et al*. identify many factors for the success in Israel´s roll out of COVID-19 vaccines, including the organizational and logistic capacities of community-based healthcare providers; the tradition of effective cooperation between various sectors particularly during national emergencies; the existence of well-functioning frameworks for making decisions about vaccinations and support tools for assisting in the implementation of vaccination campaigns; the rapid mobilization of special government funding for vaccine purchase and distribution, and timely contracting for a large amount of vaccines relative to Israel´s population [24].

The local factors influencing countries´ equitable access to vaccines include a country´s regulatory approval, the ability to implement a mass vaccination program, as well as the management of logistics and the administrative elements of mass vaccination efforts [22]. Countries in the African Region have long-standing experience conducting wide age-range mass vaccination activities, where in some cases, almost half the population (persons under 15 years of age) gets targeted in an intensive and highly coordinated manner within less than 10 days. Yellow fever and Meningitis preventive catch-up Supplementary Immunization Activities (SIAs) target even more wider age categories. Mass vaccination activities require intensive mobilization of human resource, logistical and financial resources to meet very clear objectives [25]. The planning and preparations to implement mass vaccination campaigns typically require at least 9 months, and include microplanning at the community, health facility and district levels, the training of health workers and intense social mobilization [19]. However, COVID-19 vaccination was implemented quite differently, with an initial phased approach which lasted many months, mainly due to the limited availability of vaccines, limited availability of operational funds, and a gradual expansion of the target groups.

A very small number of countries shared information on the number of vaccinated health workers, persons with co-morbidities and refugees. Similarly, not all countries have made available the data disaggregated by gender. From the available information, we note a discrepancy in coverage between the sexes in some countries in the Region. US data published in May 2021 shows that vaccination coverage was lower among adults living in counties with per-capita income less than the median [26]. In another study, there was a significant gap in coverage between different racial/ ethnic groups, but not across age groups or by sex [27].

With the reporting of AEFI data, we note gaps in the capture of serious AEFI cases in the detailed AEFI database in this study. In addition, there is a wide discrepancy in reporting rates between countries, which reflects the gaps in the reporting systems utilized, differences in the definition of what needs to be reported to the national level, challenges with accurate diagnosis, or gaps in overall completeness of reporting. However, the pattern of symptoms reported are similar to other reports from the US and Pakistan [28,29]. Previous reports have highlighted the barriers to routine reporting of AEFI which include lack of reporting tools, poor health care worker understanding of AEFI, weak or poorly coordinated reporting systems, and health care worker fear of punishment [30].

At least 26 countries conducted Intra-action reviews (IARs) to help them identify and address bottlenecks [17,31]. Most of these bottlenecks involved the vaccine supply patterns, as well as shortage of funding to cover operational costs, limiting countries´ ability to mount large scale mobilization of efforts. Countries also identified hesitancy to COVID-19 vaccination as an important challenge. An early study across multiple African countries demonstrated hesitancy among the general public, with some variety in acceptance rates across different countries [32]. Hesitancy among health workers has been documented in a number of countries [33,34].

With the ever-mutating nature of rumors and myths surrounding COVID-19 vaccines, there is a need for regular social listening, and monitoring of public perceptions in order to guide demand generation efforts in a more evidence-driven and nuanced manner. Such nuances may involve targeted multi-channel messaging to rapidly respond to concerns, as well as tailoring vaccination service delivery models to make it easier for under-vaccinated population segments to access vaccines. Our observations and the IARs indicate significant inequities of service availability and coverage within countries. National immunisation programs should regularly conduct disaggregated analysis to identify specific geographic areas and population groups to address. This will require investments in health worker capacity and tools to ensure good quality data, i.e., complete and granular data capture at the operational level, data made available timely for decision making at all levels.

The findings in this study are subject to some limitations. The dataset we analyzed had gaps in terms of completeness. The number of received doses of vaccines that countries have reported from the various sources may not be up-to-date. There have been documented backlogs of coverage data reporting from the district level in a number of countries, sometimes taking days before being entered into electronic data capture systems. The gaps in reporting of disaggregated coverage among health workers, persons with co-morbidities, and among women is another example of incompleteness. Similarly, with the low rates of reporting of AEFI cases and the wide discrepancies in reporting rates among countries, it is evident that some national AEFI surveillance systems have not been able to capture a significant proportion of cases. This study did not try to look at subnational level coverage patterns.

## Conclusion

COVID-19 vaccination coverage remains very low in the African Region, with only 5 of 46 countries attaining the target of 40% coverage by the end of 2021. Significant gaps remain in terms of the populations to be reached, in order to attain the proposed 70% target by June 2022. But, more importantly, countries will need to develop more realistic targets based on their own contexts and current levels of coverage. Countries, donors and partners should mobilized political will and resources towards the scaling up of vaccination efforts to this end. Countries should utilize tailored approaches to reach high risk and unreached populations. The emphasis should be on high quality planning and logistical organization, as well as the use of evidence to guide the program. Moreover, the reporting gaps noted in 2021 indicate the need to invest in efforts to improve the capture, analysis and use of more granular program data for decision-making.

### What is known about this topic

- Various facilities have been set up to help developing countries get access to COVID-19 vaccines;
- Equitable access to COVID-19 vaccines is critical in order to bring the COVID-19 pandemic to an end;
- The World Health Organization proposed national COVID-19 vaccination coverage target of 40% by the end of 2021.

### What this study adds

- Following a period of limited vaccine supply, the access to COVID-19 vaccines has improved since August 2021, along with modest increase in the weekly rates of coverage across the African Region;
- COVID vaccination coverage remains very low in the WHO African Region with only 6.8% of the population having received full vaccination, and only 5 countries reaching the target of 40% coverage by December 2021;
- AEFI reporting rates remain very low, and show wide discrepancy across countries, indicating the need for standardizing reporting systems. Significant gaps exist in terms of the quality of reporting as well as sharing of coverage and vaccine safety data from the countries in the WHO African Region.
